# Suppressor of cytokine signalling gene expression is elevated in breast carcinoma

**DOI:** 10.1038/sj.bjc.6601115

**Published:** 2003-07-29

**Authors:** M Raccurt, S P Tam, P Lau, H C Mertani, A Lambert, T Garcia-Caballero, H Li, R J Brown, M A McGuckin, G Morel, M J Waters

**Affiliations:** 1 CNRS UMR 5123, Bât. Raphael Dubois, Université Claude Bernard-Lyon 1, 43 Blvd 11 Novembre 1918, F69622 Villeurbanne cedex, France; 2 School of Biomedical Sciences and Institute for Molecular Bioscience, University of Queensland, St Lucia, Queensland 4072, Australia; 3 Departamento de Ciencias Morfológicas, Facultad de Medicina, Universidad Santiago de Compostela, c/San Francisco s/n, Santiago de Compostela 15705, Spain; 4 Mater Medical Research Institute, Level 3, Aubigny Place, Mater Misericordiae Hospital, S. Brisbane, Queensland 4101, Australia

**Keywords:** cytokine, SOCS, breast, prolactin, growth hormone, carcinoma

## Abstract

Cytokines are important for breast cell function, both as trophic hormones and as mediators of host defense mechanisms against breast cancer. Recently, inducible feedback suppressors of cytokine signalling (SOCS/JAB/SSI) have been identified, which decrease cell sensitivity to cytokines. We examined the expression of SOCS genes in 17 breast carcinomas and 10 breast cancer lines, in comparison with normal tissue and breast lines. We report elevated expression of SOCS-1–3 and CIS immunoreactive proteins within *in situ* ductal carcinomas and infiltrating ductal carcinomas relative to normal breast tissue. Significantly increased expression of SOCS-1–3 and CIS transcripts was also shown by quantitative *in situ* hybridisation within both tumour tissue and reactive stroma. CIS transcript expression was elevated in all 10 cancer lines, but not in control lines. However, there was no consistent elevation of other SOCS transcripts. CIS protein was shown by immunoblot to be present in all cancer lines at increased levels, mainly as the 47 kDa ubiquitinylated form. A potential proliferative role for CIS overexpression is supported by reports that CIS activates ERK kinases, and by strong induction in transient reporter assays with an ERK-responsive promoter. The *in vivo* elevation of SOCS gene expression may be part of the host/tumour response or a response to autocrine/paracrine GH and prolactin. However, increased CIS expression in breast cancer lines appears to be a specific lesion, and could simultaneously shut down STAT 5 signalling by trophic hormones, confer resistance to host cytokines and increase proliferation through ERK kinases.

Cytokines are key regulators of mammary epithelial cell function, exerting regulatory actions on proliferation, differentiation, apoptosis and immune surveillance. For example, mammary ductal proliferation and branching is promoted by growth hormone (Silberstein and Daniel, 1987), while alveolar cell proliferation and secretory function require the presence of prolactin (Vonderhaar, 1987). Epidemiologic evidence indicates a role of prolactin in both the pathogenesis and progression of breast cancer (Hankinson *et al*, 1999). However, both of these cytokine hormones can promote proliferation of breast cancer lines (Fuh and Wells, 1995; Kaulsay *et al*, 1999) and their receptors are expressed in a wide variety of breast cancers (Clevenger *et al*, 1995; Mertani *et al*, 1998). Both can also be synthesised locally in the mammary gland (Clevenger *et al*, 1995; Reynolds *et al*, 1997), and antagonists of the prolactin and growth hormone (GH) receptors are able to inhibit proliferation of breast cancer lines (Fuh and Wells, 1995). Moreover, autocrine production of GH in human mammary cell lines promotes the transformed phenotype (Kaulsay *et al*, 1999), and prolactin increases the motility of breast cancer lines (Maus *et al*, 1999).

Cytokine receptors exert their actions through activation of the Janus kinases (JAKs), which tyrosine phosphorylate both the receptors and their downstream signalling targets, notably the STAT family of transcriptional activators (Argetsinger and Carter-Su, 1996). The major STATs responsible for regulation of the mammary gland by GH and prolactin are STAT 5a and 5b (Teglund *et al*, 1998), both receptors are also able to activate STAT 3 (Argetsinger and Carter-Su, 1996). Immune cytokines such as the interferons also utilise JAKs, and play an important role in mediating the host response to development of breast cancer (Allione *et al*, 1994; Camp *et al*, 1996; Tannenbaum and Hamilton, 2000).

Recently, it has been shown that cytokine signalling through the JAK/STAT pathway is controlled by a classical feedback loop through suppressors of cytokine signalling (SOCS/JAB/SSI) (Hilton, 1999). Suppressors of cytokine signalling proteins are rapidly induced by activated STATs and act to block the cytokine signal either by direct inhibition of JAKs (SOCS-1), by binding to tyrosine phosphorylated receptor so as to exclude binding of other SH2 and PTB domain-containing signalling proteins such as STATs (CIS), or by both mechanisms (SOCS-3) (Ram and Waxman, 1999). SOCS proteins are also able to accelerate proteasome-mediated destruction of the activated cytokine–receptor complex (Ram and Waxman, 2000; Kamizono *et al*, 2001), particularly in the case of CIS. These inhibitory regulators not only attenuate the signal from the activated cytokine receptor itself, but can also decrease cell sensitivity to other cytokines and hormones, such as insulin (Emanuelli *et al*, 2000). Thus, prolactin, the major trophic cytokine for the breast, is known to rapidly induce SOCS-1–3 and CIS, and this results in loss of sensitivity to subsequent prolactin challenge (Tam *et al*, 2001). SOCS-1 has been shown to be critical for preventing interferon *γ* (IFN*γ*)-mediated growth arrest of M-1 myeloid cells (Sakamoto *et al*, 1998), and overexpression of SOCS-1 or SOCS-3 in breast cancer lines inhibits the antiviral and antiproliferative ability of IFN*γ* (Song and Shuai, 1998). Interestingly, there is evidence that mammary carcinoma lines are resistant to the growth inhibitory actions of IFN*γ* (Harvat and Jetten, 1996), which could be the result of constitutive expression of SOCS proteins. Indeed, constitutive SOCS-3 expression has been reported for cutaneous T-cell lymphoma lines, and reversal of this expression was associated with increased sensitivity to interferon *γ* inhibition of proliferation (Brender *et al*, 2001).

In view of the importance of SOCS expression in regulation of mammary cell function, and its potential importance in breast cancer, we have examined the expression of SOCS-1–3 and CIS in both breast cancers and in breast cancer lines. The former provides information about SOCS expression *in vivo* in the context of host responses, while the latter allows for demonstration of constitutive expression that may result from genomic alteration.

## MATERIALS AND METHODS

### Tissue samples

Surgical samples of breast cancer were selected from the files of University Clinical Hospital of Santiago de Compostela (Pr. Forteza) (Spain), which had been collected with Institutional Ethics Committee approval. A total of 20 human breast samples defined by standard histopathological criteria were studied: three normals, six *in situ* ductal carcinomas without lymph node metastasis, and 11 infiltrating ductal carcinomas. Tissues were fixed in 10% buffered formalin for 24 h at room temperature, dehydrated, embedded in paraffin and sectioned by standard procedure. Sections of 5 *μ*m thick were mounted on sterilised 3-aminopropyl-triethoxysilane-coated slides (Sigma, St Louis, MO, USA) and dewaxed before processing for *in situ* hybridisation or immunohistochemistry.

### Cell lines

The BT-20, MA-11, MDA-MB-468, SK-BR-3, KPL-1, T-47D, UACC-893, ZR-75-1 and ZR-75-30 cell lines were obtained from American Type Culture Collection (Manassas, VA, USA) and grown in RPMI 1640 medium supplemented with 10% FCS. The HMEC184, MCF-10A and MCF7 cell lines were gifts from Dr R Sutherland (Garvan Institute, Sydney, Australia). The HMEC184 cell line was grown in MCDB 170 culture medium supplemented with bovine pituitary extract (Gibco BRL, Rockville, MD, USA) and 5 *μ*g ml^−1^ gentamycin. The MCF-10A cell line was grown in DMEM: Hams F12 (1 : 1) supplemented with 20 ng ml^−1^ EGF, 10 *μ*g ml^−1^ insulin, 500 ng ml^−1^ hydrocortisone, 2.5 mmol l^−1^ L-glutamine, 5% horse serum and 5 *μ*g ml^−1^ gentamycin. The MCF7 cell line was cultured in RPMI 1640 medium supplemented with 10% Serum Supreme (a fetal bovine serum alternative supplied by Biowhittaker, USA) and 5 *μ*g ml^−1^ gentamycin.

### Probes and antibodies

Full-length cDNA probes were used to detect SOCS 1, 2 and 3, and CIS mRNAs (Starr *et al*, 1997). The human prolactin receptor probe was directed to the extracellular domain, nucleotides 346–1001 (Boutin *et al*, 1989). The human GH receptor probe was also directed to the extracellular domain, nucleotides 1–720 (Godowski *et al*, 1989). These probes were labelled with *α*[^35^S]dATP (NEN Life Sciences, Boston, MA, USA) by random priming and then purified from the free nucleotides with Nick Columns (Amersham Pharmacia Biotech AB, Sweden) according to the suppliers' instructions.

Goat antibody raised against a peptide corresponding to the carboxy terminal sequence of human SOCS-1 (cat. no. sc-7005, C20) and to the amino-terminal sequence of human CIS (cat. no. sc-1529, N-19) were purchased from Santa Cruz Biotechnology (Santa Cruz, CA, USA). Rabbit anti-CIS antibody was a generous gift of Professor Yoshimura, Institute of Life Science, Kurume, Japan (described in Matsumoto *et al* (1997)). Rabbit anti-SOCS-3 antibody was raised against murine SOCS-3, and was a generous gift of Dr Doug Hilton (Walter & Eliza Hall Institute, Parkville, Australia). The streptavidin–avidin–peroxidase complex procedure was employed with diaminobenzidine as chromogen (Duet kit, Dakopatts, Carpinteria, CA,USA).

### *In situ* hybridisation

Dewaxed sections were digested with 5 *μ*g ml^−1^ proteinase K (Roche Diagnostics, Meylan, France) in a Tris (20 mmol l^−1^)-CaCl_2_ (2 mmol l^−1^) buffer for 30 min at 37°C. The slides were dehydrated in ethanol series and air-dried. Sections were then covered with hybridisation buffer containing 50% deionised formamide, 10% dextran sulphate, 4 × standard saline citrate (SSC) (1 × SSC=0.15 mol l^−1^ NaCl, 0.015 mol l^−1^ sodium citrate, pH 7.0), 1 × Denhardt's solution (50 × Denhardt's solution=1% BSA, 1% Ficoll 400, 1% polyvinylpyrolidone), 100 *μ*g ml^−1^ yeast transfer RNA, 10 mM dithiothreitol and labelled probe (5000 d.p.m. *μ*l^−1^ corresponding to 0.1–0.5 *μ*g ml^−1^ of hybridisation buffer). *In situ* hybridisation was performed overnight at 40°C. Sections were washed sequentially in 2 × SSC for 1 h at room temperature, then for 1 h at 45°C and subsequently in 1 × SSC, followed by 0.5 × SSC and 0.1 × SSC, each for 30 min at room temperature. For macroautoradiography, dehydrated sections were apposed onto autoradiographic films (Hyperfilm [^3^H], Amersham-Pharmacia, Orsay, France) for 1 week at room temperature. For microautoradiographical purposes, the slides were dipped in NTB2 nuclear emulsion (Kodak, Paris, France), exposed at 4°C for 30 days, developed in D19 (Kodak), and finally counterstained with eosin–haemalum. Slides were observed under a fluorescent light microscope by epipolarisation. Controls for the specificity of the *in situ* hybridisation included: (1) omission of the probe; (2) hybridisation with a heterologous labelled POMC cDNA probe, (3) hybridisation with undenaturated labelled cDNA probe and (4) excess of nonlabelled probe.

### Semiquantification of the *in situ* hybridisation

A semiquantitative analysis of gene expression was performed as previously described (Peyrol *et al*, 1997; Mertani *et al*, 1998) on more than six macroautoradiograms from each sample obtained under similar conditions. Sections were run through the same hybridisation, washing, and detection assays in order to render the signal levels comparable for each probe. The levels of mRNA were analysed in histopathological structures identified from contiguous sections stained with classical haematoxylin/phloxine/saffron (HPS). We quantified the grey level obtained on film in two compartments of normal tissues (duct and surrounding normal connective tissue), in infiltrating ductal breast carcinomas (cancerous area and normal connective tissue) and in three compartments of *in situ* ductal breast carcinomas (cancerous duct, epitheliostromal interface and normal connective tissue). Normal connective tissue was analysed as far away as possible from the tumor. Autoradiograms were analysed under standardised conditions, using a densitometric computer imaging system (Leica, Lyon, France). Optical densities for each sample were measured, in homogeneous areas (5–15/autoradiogram, excluding artefacts) and then averaged. Background signal was measured on negative control samples and substracted from each measure. A linear relationship (standard curve) with a slope depending on exposure time, was found by single regression analysis between optical density values of the standards and their corresponding radioactivity. It was then possible to compare the different radioactivity values for each probe used, which were expressed in arbitrary units, by inserting the optical density value of each sample into the standard curve equation. The levels of the signals obtained after hybridisation were expressed as the mean±s.e. Statistical analysis was performed using one-way analysis of variance, followed by Student's *t*-test. Differences were considered significant at *P*<0.05.

### Northern Hybridisation

Total RNA was isolated from cell lines using TRIzol Reagent (Gibco BRL, Rockville, MD, USA) according to the manufacturer's protocol. Aliquots of 20 *μ*g total RNA were denatured and subjected to electrophoresis on a 1.2% agarose, 11% formaldehyde gels, then transferred to HyBond N membrane (Amersham Pharmacia Biotech, Buckinghamshire, UK). RNA was stabilised by UV-crosslinking and baking for 30 min at 80°C. ^32^P-labelled cDNA probes specific for SOCS-1, -2, -3 and CIS were prepared by the random prime labelling system, Rediprime II (Amersham Pharmacia Biotech, UK) and then purified from the free nucleotides with Nick Columns (Amersham Pharmacia Biotech AB, Sweden) according to the suppliers' instructions.

The blots were prehybridised with NorthernMax prehyb/hyb Buffer (Ambion Inc., Austin, TX, USA) for 4 h at 50°C before a ^32^P-labelled probe was added. Blots were then hybridised for 16 h at the same temperature. After hybridisation, the blots were washed in 2 × SSC/0.1% SDS at 65°C for 30 min, twice. Autoradiography was then carried out at −70°C using Super HR-G30 X-Ray Film (Fuji Photo Film, Tokyo, Japan). Blots were finally stripped and rehybridised with a ^32^P-end-labelled oligomer probe specific for 18S ribosomal RNA to ensure equal loading.

### Immunoblot

Confluent cultures of breast cancer lines were scraped and homogenised on ice in RIPA buffer (150 mmol l^−1^ NaCl, 1% NP40, 0.5% sodium deoxycholate, 0.1% SDS, 50 mM Tris (pH 7.5)) with Complete protease inhibitor cocktail (Roche Molecular Biochemicals, Mannheim, Germany; cat 1697498). Lysates were then homogenised with a Polytron homogeniser for four 15 s pulses at maximum speed on ice, boiled for 10 min with 0.5 vol of 3 × Laemmli sample buffer and centrifuged at 15 000 r.p.m. for 20 min. Protein samples (100 *μ*g) were immunoblotted as previously described (Tam *et al*, 2001). After semidry transfer, nitrocellulose membranes were blocked with TBS (pH 8.0) containing 5% skim milk, 0.1% Tween 20 and probed with goat anti-CIS antibody (1 : 250, Santa Cruz Biotechnology; cat. sc-1529) at 4°C overnight. Thereafter, membranes were incubated with HRP conjugated rabbit anti-goat antibody (Pierce, Rockford, IL, USA) at 1 : 16 000 for 1 h at room temperature followed by development with West Pico Chemiluminescence Substrate (Pierce, Rockford, IL, USA).

### Immunohistochemistry

Sections were dewaxed, rehydrated and pretreated with 3% H_2_O_2_. They were then blocked with 10% normal horse serum, followed either by 1 : 500 rabbit anti-CIS antiserum (a gift from A Yoshimura) or 1 : 500 rabbit anti-SOCS anti-serum (a gift of Dr D Hilton) or 1 : 500 non-immune rabbit serum (as negative control), overnight at 4°C. Thereafter, sections were incubated with 1 : 200 biotinylated donkey anti-rabbit IgG (cat. RPN1004, Amersham, UK) for 2 h at room temperature, followed by 1 : 200 streptavidin–biotinylated HRP complex (cat. RPN 1051, Amersham, UK) for 2 h at room temperature. The signal was visualised by incubation with diaminobenzidine substrate for 5 min. Sections were counterstained with haematoxylin and dehydrated before mounting.

### ERK reporter assay

This was carried out in CHO cells as described in Clarkson *et al* (1999) using a luciferase coding sequence downstream of an egr-1 promoter fragment comprising 624 bp 5′ of the initiation codon. CHO K1 cells seeded at 3 × 10^5^ cells per well of a six-well plate and were transfected with DOTAP at a final DNA concentration of 4 *μ*g per well. This comprised 1 *μ*g of the reporter construct and 0, 3, 10, 30, 100, 300, 1000 and 3000 ng of the CIS expression vector (pCDNA3). Empty vector was added to make the total up to 3 *μ*g in all cases. The medium was changed at 18 h, the cells were then left for 48 h in 0.125% serum supreme (Biowhittaker, Walkersville, MD, USA) in Hams' medium before harvesting for luciferase assay as described in Clarkson *et al* (1999).

## RESULTS

### Breast cancer tissues

#### *In situ* hybridisation: Overview

**Figure 1:**
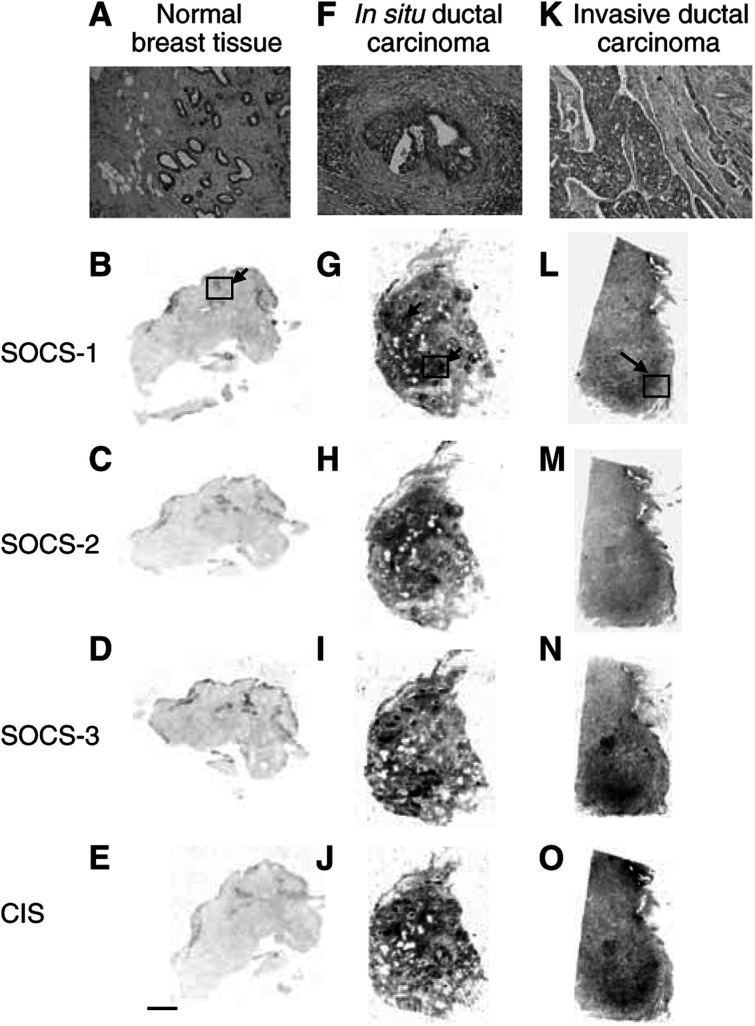
Macroautoradiographic pattern of ISH of SOCS-1–3, CIS mRNA performed on sections of normal breast (**A**–**E**) and typical lesions of *in situ* (**F**–**J**) and invasive (**K**–**O**) ductal carcinomas. Semiquantitative expression of mRNA was performed on typical areas of the samples, as illustrated in **B**, **G** and **L**, after precise microscopic examination of the contiguous HPS-stained sections (**A**, **F** and **K**). The signal (dark areas) corresponds to different levels of SOCS and CIS genes expression. On adjacent sections of normal breast tissue, the signal obtained for the four genes is localised to the ducts and lobules (arrow) (**B**–**E**). On adjacent sections of *in situ* ductal carcinoma, the same intense signal is observed for the four genes and is localized to areas corresponding to enlarged ducts and periductal stroma reaction (arrows) (**G**–**J**). On adjacent sections of invasive ductal carcinoma, the increased density observed with the four probes encompasses the area corresponding to tumour cells infiltrating the cellular stroma (**L**–**O**). Thus, a more intense signal is seen specifically localised to the area of tumour invasion (arrow). Bar, 5 mm.

**Figure 2:**
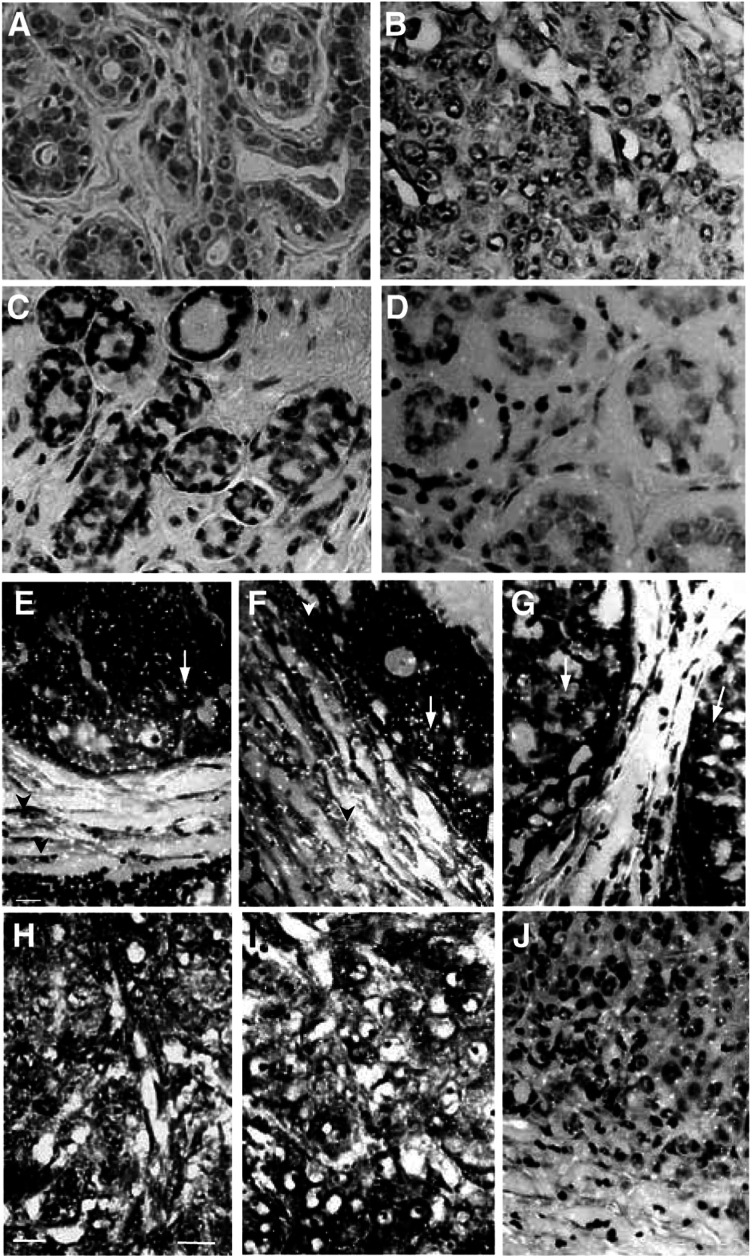
Cellular expression of SOCS-1–3 and CIS mRNA in normal breast (**A**, **C**, **D**), *in situ* (**E**–**G**) and invasive (**H**–**J**) ductal breast carcinomas, evidenced by the presence of bright silver grains on emulsion-coated sections. On sections of normal breast tissue, basal levels of SOCS-1 (**C**) and CIS (**D**) were detected in normal epithelial cells and in scattered fibroblasts of the surrounding connective tissue. On sections from patients with *in situ* ductal carcinoma, expression of SOCS-1 (**E**), SOCS-2 (**F**) and CIS (**G**) transcripts was strongly associated with proliferative tumour cells (arrows) of the enlarged ducts, in concentric layers of fibroblastic cells (arrowheads) and in lymphocytes of inflammatory infiltrates (*). On sections from patients with invasive ductal carcinoma, gene expression of SOCS-1 (**H**), SOCS-3 (**I**) and CIS (**J**) was abundantly detected in the whole area of the tumour. The close association of cancerous cells and stromal cells prevents the precise identification of the positive cell component. No signal was observed when *in situ* hybridisation was performed with heterologous cDNA probe as a negative control on normal breast tissue (**A**) and invasive ductal carcinoma (**B**). Bar, 50 *μ*m.

**Figure 3:**
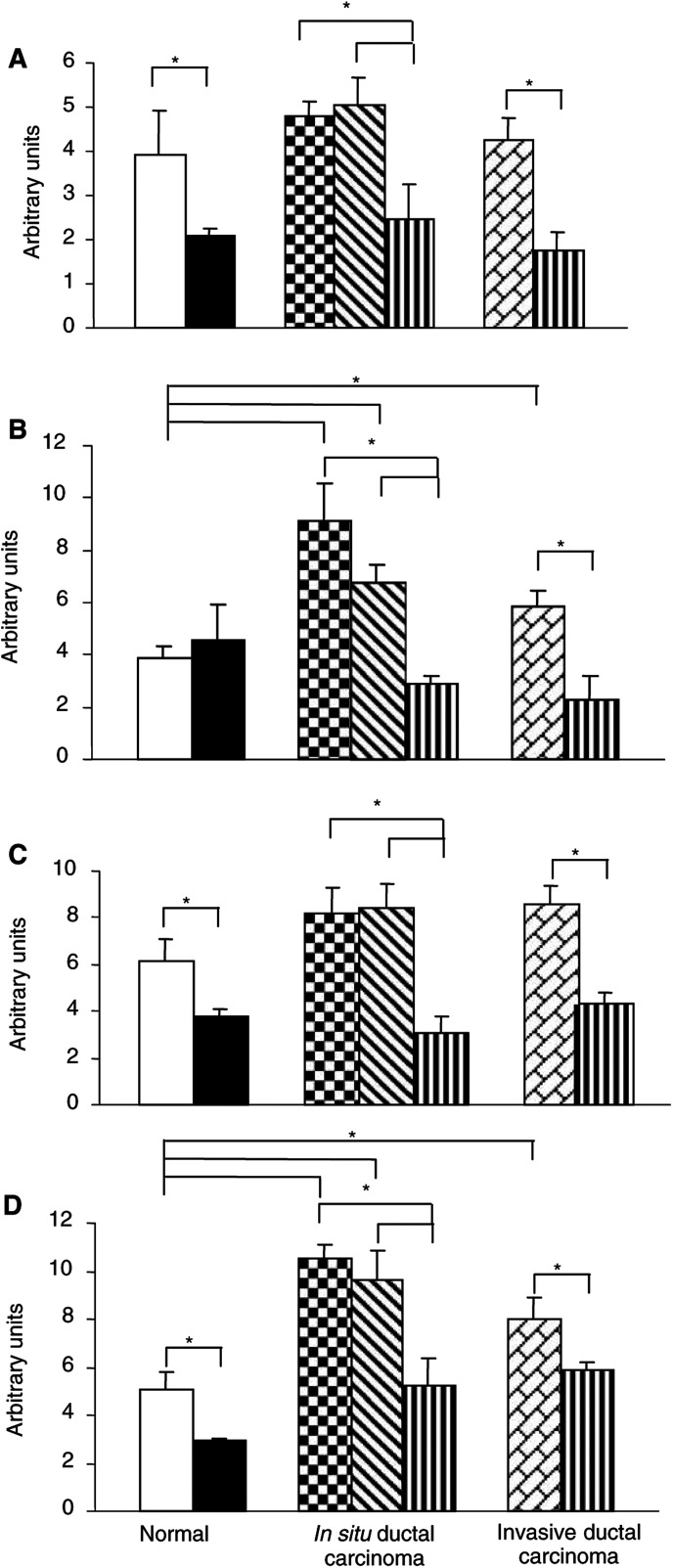
Quantification of mRNA levels was performed on macroautoradiograms from normal breast, *in situ* and invasive ductal carcinomas as described in Materials and Methods. Optical densities were measured for the four probes (**A**: SOCS-1, **B**: SOCS-2, **C**: SOCS-3, **D**: CIS) in typical areas according to pathological criteria: (□), normal duct; (▪), normal connective tissue; (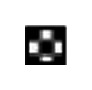), cancerous ducts; (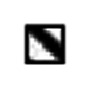), reactive stroma; (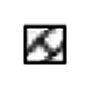), invasive area; (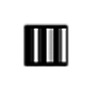), adjacent normal connective tissue; expressed in arbitrary units±s.e.m. ^*^P<0.05.

Gene expression of different SOCS and CIS mRNAs in breast disorders was visualised through hybridisation between labelled probe and target mRNA, apparent as variable grey levels on film (Figure 1

) and bright silver grains on tissue sections (Figure 2

). The results obtained were always qualitatively consistent. The specificity of the signal was demonstrated by use of the controls described in the Materials and Methods. Thus, no specific signal could be detected after hybridisation with unrelated labelled cDNA probe or when cold probe was used as competitor (Figure 2A, B). Expression of the four genes was localized to identical areas but with variable intensity as shown by the quantitative analysis (Figure 3

). Consequently, only typical results are shown (Figures 1 and 2).

#### Regional expression

In normal breast tissue obtained at a distance from the tumour, the macroautoradiographic hybridisation signal for SOCS-1–3 and CIS mRNA was observed in regions corresponding to epithelial component of the duct (Figure 1B–E). A weak, homogeneous signal associated with the whole connective tissue area appeared to be specific in comparison to control sections hybridised with unrelated cDNA probe (not shown) and presumably represents the physiological level of SOCS-1–3 and CIS expression.

Higher expression of SOCS-1–3 and CIS genes was evidenced in ducts enlarged by tumour cell proliferation and in the periductal reactive stroma. On sections obtained from patients with *in situ* breast carcinoma, this signal decreased in connective tissue located at a distance from the cancerous ducts (Figure 1G–J). In sections obtained from patients with infiltrating breast carcinoma, SOCS-1–3, and CIS transcript expression was stronger in the region corresponding to the most active zone of the tumour (Figure 1L–O).

#### Semiquantitative analysis of mRNA

Relative level of SOCS-1 (Figure 3A), SOCS-2 (Figure 3B), SOCS-3 (Figure 3C) and CIS (Figure 3D) mRNA expression was measured densitometrically on autoradiographic films on normal and pathologic breast sections, in areas selected as defined in Materials and Methods. Note that the signal intensities obtained for each mRNA cannot be compared, since the four different probes did not hybridise with the same efficiency.

In normal breast tissue, the hybridisation signal was stronger in the epithelial ducts than the connective tissue, and this was seen for all probes except SOCS-2 (Figure 3A–D). On the pathologic sections, we consistently observed that SOCS-1–3 and CIS gene expression was strongly associated with the tumor cells and was significantly higher than the basal level in normal adjacent epithelial and connective tissues (*P*<0.05).

SOCS-1 gene expression increased in the cancerous ducts (+22% *vs* normal ducts, *P*<0.05) and in the reactive stromal area (+104% *vs* adjacent connective tissue, *P*<0.01) of sections obtained from patients with *in situ* ductal carcinoma (Figure 3A). SOCS-2 gene expression more significantly increased in the cancerous cells (+135% *vs* normal duct, *P*<0.05) of *in situ* ductal carcinoma, as well as in the tumoral area of infiltrating carcinoma (+50% *vs* normal duct, *P*<0.05) (Figure 3B). SOCS-3 gene expression significantly increased in the cancerous ducts (+32% *vs* normal duct, *P*<0.05) and in the reactive stromal area (+170% *vs* adjacent connective tissue, *P*<0.01) of *in situ* ductal carcinoma, as well as in tumour area of sections obtained from patients with infiltrating carcinoma (+39% *vs* normal duct, *P*<0.05) (Figure 3C). CIS gene expression was strongly increased in tumour cells (+120% *vs* normal ducts, *P*<0.01) of *in situ* ductal carcinoma, as well as in tumour area of sections obtained from patients with infiltrating carcinoma (+60% *vs* normal ducts, *P*<0.05). CIS expression was also elevated in adjacent reactive stroma relative to normal tissue (Figure 3D).

#### Cellular patterns of SOCS-1–3 and CIS gene expression

To determine which cell components expressed SOCS-1–3 and CIS mRNA in normal and pathologic human breast, microautoradiographic methodology was utilized as previously described (Mertani *et al*, 1998). Basal levels of SOCS-1 (Figure 2C), SOCS-2, -3 (not shown) and CIS (Figure 2D) mRNA were detected in both luminal epithelial and myoepithelial cells of normal ducts and in scattered fibroblasts of the surrounding normal stroma.

In sections from patients with intraductal breast carcinoma, the expression of the four genes was localised to cancerous cells as well as to cells of the reactive stroma surrounding the ducts (Figure 2E–G), as was evident with the macroautoradiographic study described above (Figure 1G–J). In this reactive stroma, concentric layers of myoepithelial and myofibroblastic cells showed an intense signal. The stroma was often infiltrated by numerous inflammatory cells which were also strongly labelled (Figure 2E). As reflected by the semiquantitative data (Figure 3), there was no difference in signal intensity between the signal obtained in the tumour cells and the periductal reactive stroma (Figure 2E–G).

In sections obtained from patients with infiltrating ductal breast carcinoma, gene expression of SOCS-1, -2, -3 and CIS was associated with the entire area of the tumour invasion (Figure 2H–J). The tumour architecture and the stromal disorganization of this type of breast cancer limited clear distinction between silver grains associated with tumour cells and stromal cells accompanying the tumour progression.

#### Immunohistochemistry

**Figure 4:**
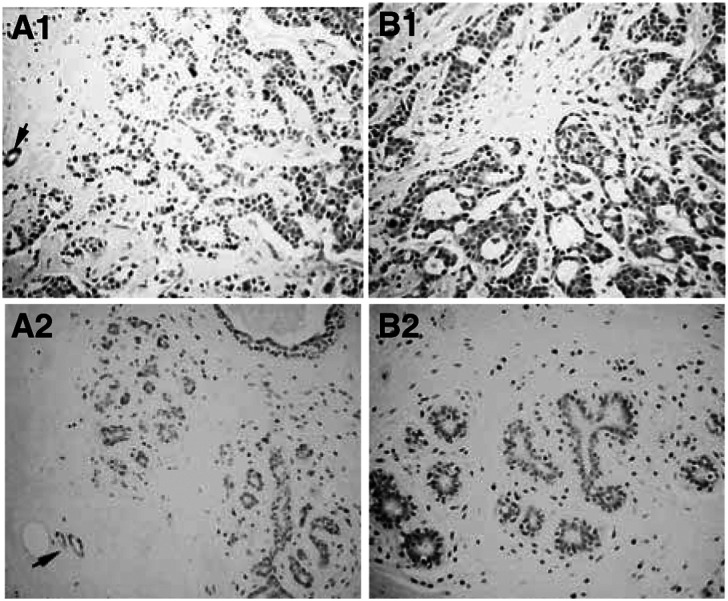
Immunostaining for CIS and SOCS-3 in normal breast tissue and infiltrating ductal carcinoma. Tissues were fixed and processed as described in the Materials and Methods section. Cancerous cells were strongly immunoreactive for CIS (**A1**) and SOCS-3 (**B1**) when compared with normal tissues (**A2** and **B2**, respectively). Arrows indicate equal immunoreactivity in blood vessels of normal and cancerous tissue. Bar, 100 *μ*m.

CIS immunoreactivity was weak in normal duct luminal epithelium and myoepithelial cells, positive in blood vessels, and strongly positive in carcinoma (Figure 4

, panels A1 and A2). Fibroblasts of connective tissue did not show significant CIS immunoreactivity. Immunoreactivity for SOCS-3 was similar to that for CIS, with carcinoma staining more prominent than in normal epithelium, although staining in blood vessels was not as evident (Figure 4, panels B1 and B2). Both in the case of CIS and for SOCS-3, no immunoreactivity was seen when nonimmune serum was run in parallel sections (not shown).

### Breast cancer lines

#### Northern analysis for SOCS transcripts

**Figure 5:**
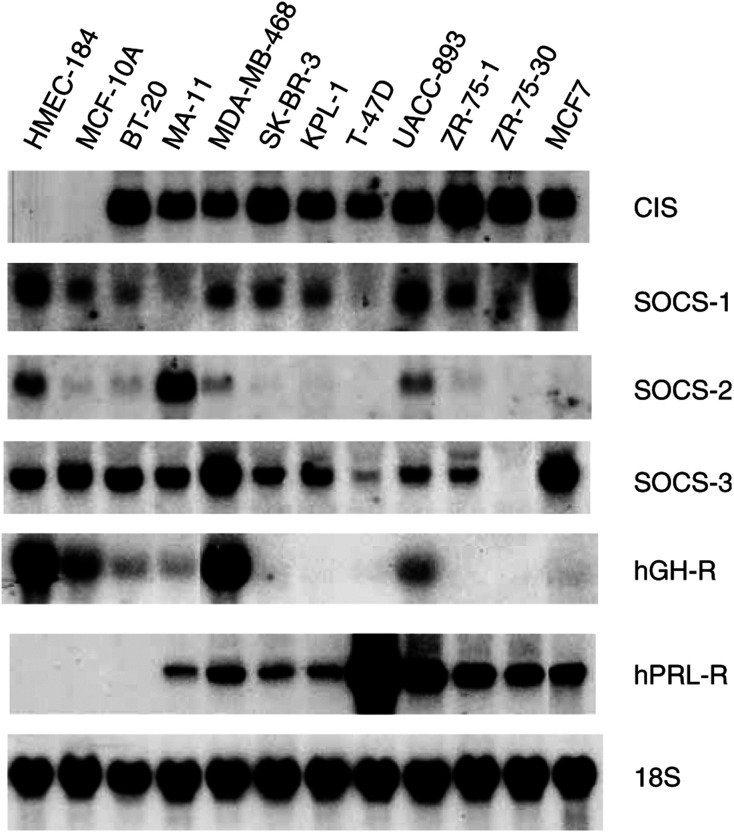
Expression of transcripts for CIS and SOCS-1–3 and for GH and prolactin receptors in normal (HMEC184 and MCF-10A) and 10 breast cancer lines. Total RNA was extracted from cell lines, subjected to electrophoresis on a formaldehyde gel and blotted on a nylon membrane. Identical blots were run and each was hybridized with a specific cDNA probe. The blots were then washed and exposed to X-ray films as described in Materials and Methods. The exposure time for CIS was 2 days; for SOCS-1, 5 days; for SOCS-2, 5 days; and for SOCS-3, 3 days. Blots were stripped and reprobed for 18S rRNA to ensure equal loading.

A survey of 10 breast cancer lines for SOCS-1–3 and CIS transcripts showed that only CIS mRNA was elevated in all transformed lines in comparison with the two phenotypically normal lines, HMEC184 and MCF-10A. There was no correlation between CIS transcript expression and GH receptor mRNA expression across these lines (Figure 5

). Prolactin receptor mRNA was expressed in all but one of the transformed lines and not in the two control lines, but the level of prolactin receptor expression did not correlate with the level of CIS mRNA across these lines (Figure 5). It is possible that endogenous or exogenous prolactin could elevate CIS transcripts in these lines, but the lack of correlation between CIS transcript levels and expression of other SOCS transcripts argues against this, given that prolactin is able to induce SOCS-1,-3 and CIS transcripts in mammary tisue (Tam *et al*, 2001).

#### Immunoblot analysis

**Figure 6:**
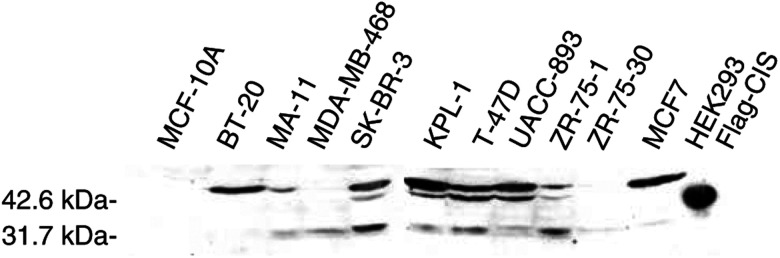
CIS protein expression in breast cancer lines. Total cell lysate was obtained from confluent cultures of breast cancer lines or normal lines. Lysates were then immunobloted and probed with goat anti-CIS antibody as described in the Materials and Methods. FLAG-tagged CIS expressed in HEK 293 cells was used as a positive control. Signal corresponding to the CIS protein was detected migrating at 32 kDa in breast cancer cell lines. Another slower migrating band at 47 kDa corresponds to the ubiquitinylated form of CIS.

Immunoblot of cell lysates from these breast cancer lines for CIS protein revealed a high level of expression of bands around 47 000 and 32 000 for all cancer lines (Figure 6

). The 47 000 bands correspond to those of the ubiquitinylated form of CIS (Tauchi *et al*, 2001), and were more intense than the 32 000 band in all cases except for MDA-MB-468 and ZR 75–30 lines. These bands were absent in the MCF-10A control line, corresponding to its lack of CIS transcripts. Note that the FLAG tagged CIS control runs more slowly because of the contribution from the tag.

**Figure 7:**
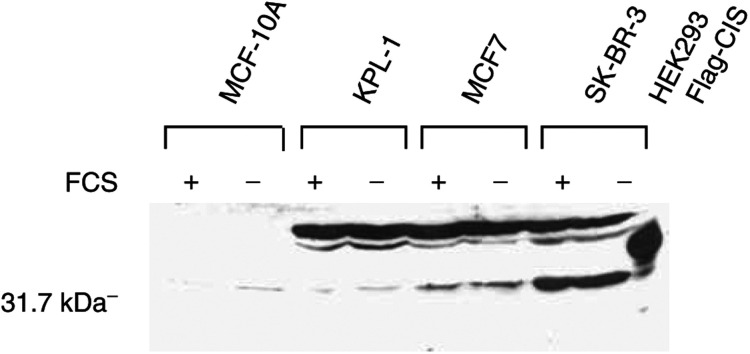
Lack of effect of serum on expression of CIS protein in breast cancer lines. Cells were exposed to 10% serum or serum starved for 12 h before harvesting for immunoblot analyses as for Figure 6.

In order to determine if expression of the CIS protein was dependent on serum factors, including prolactin and GH, the breast cancer lines were deprived of serum for 12 h before harvesting for immunoblot analysis. As can be seen in Figure 7

, serum deprivation was without effect on CIS protein expression in the lines we examined.

#### ERK reporter assay

**Figure 8:**
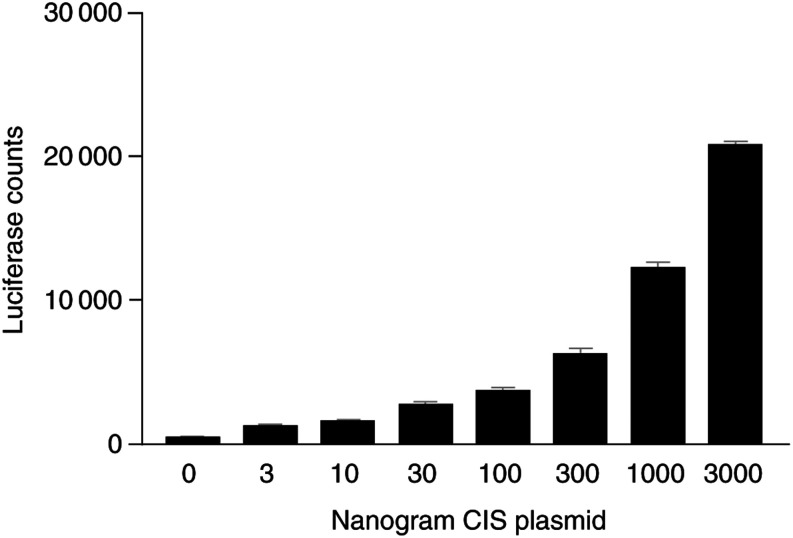
CIS transfection results in elevated ERK reporter activity. CHO cells were transfected with increasing amounts of CIS expression plasmid and an ERK reporter plasmid. The total amount of DNA was normalised to 3 *μ*g with empty pcDNA3. Luciferase assay was carried out as described in the Materials and Methods section. Luciferase counts expressed as mean±s.e.m., four replicates per point. This result was obtained on three separate occasions.

This was carried out with a luciferase reporter construct that possesses five SRF/Ets response elements, which are responsive to ERK stimulation through phosphorylation of Elk-1 and Sap1a (Gille *et al*, 1995; Janknecht and Hunter, 1997), but has no STAT5 response elements (Clarkson *et al*, 1999). As shown in Figure 8

, transfection with an increasing level of CIS expression plasmid results in increased ERK reporter activity. The effect is substantial, fold inductions at 3 *μ*g CIS plasmid being 57-, 55- and 27-fold in three independent assays.

## DISCUSSION

The present study demonstrates that SOCS-1, -2, -3 and CIS mRNAs are expressed more strongly in human breast carcinoma. We have identified the cell types expressing SOCS and CIS mRNA, and quantified the relative levels of expression in human breast tumours of increasing severity from a preinvasive stage (ductal carcinoma) to invasive carcinoma, for which we have previously documented the gene expression levels for both GH and PRL receptors (Mertani *et al*, 1998).

In the normal human breast, lower levels of SOCS and CIS mRNA were found in both ductal epithelial cells and the fibroblastic component. This level of gene expression was also found in normal tissue located at a distance from the tumour in all the pathologic samples examined. Our results show upregulation of SOCS and CIS gene expression in malignant breast disorders. In ductal breast carcinoma, SOCS-2 and CIS gene expression was particularly strong in tumour cells within the ducts, which were isolated from the reactive stroma by the myoepithelial barrier. This result can be compared to the elevated CIS gene expression level seen *in vitro* with breast cancer lines. Surrounding the cancerous ducts, stronger gene expression was also evidenced in the region of reactive stroma showing features of fibrogenesis and immune activation. High levels of SOCS-1–3 and CIS gene expression were associated with the fibroblasts, inflammatory cells and endothelial cells of the neovascularisation, which essentially comprises the reactive stroma. The local host defense is particularly important during the progression period from *in situ* to invasive ductal carcinoma and is characterised by intense cytokine production from the lymphocytic infiltration. Thus, a study by Camp *et al* (1996) performed on 89 human breast carcinomas showed strong lymphocytic production of Il-2, Il-4, TGF-*β*1, TNF-*α* as well as lower levels of IFN*γ* and GM-CSF. These cytokines are major activators of SOCS/CIS signalling, particularly SOCS-1 and -3 in granulocytes and lymphocytes (Hilton, 1999; Dogusan *et al*, 2000).

In addition to the conventional inflammatory cytokines, PRL and GH, the major mammotrophic hormones, may be involved in SOCS gene induction during breast carcinogenesis. These are also known to induce substantial SOCS-1–3 and CIS gene expression (Adams *et al*, 1998; Tam *et al*, 2001). The presence of GH and PRL receptors in both epithelial and stromal cells has been previously shown in the normal human breast and in various benign and malignant human breast disorders (Clevenger *et al*, 1995; Mertani *et al*, 1998; Gebre-Medhin *et al*, 2001). Recent studies have shown the importance of autocrine/paracrine production of hGH and hPRL during human breast carcinoma cell proliferation *in vitro* (Kaulsay *et al*, 1999; Mertani *et al*, 2001) and have proposed that local production of PRL *in vivo* is more important than systemic PRL for the tumour formation (reviewed in Wennbo and Tornell, 2000). We have demonstrated *in vivo* hGH production from human breast carcinoma and established that increased expression of the hGH gene occurs in the epithelial component and that *de novo* stromal expression of hGH is associated with the neoplastic progression of the mammary gland (Raccurt *et al*, 2002). Other studies (Hattori *et al*, 1990; Wu *et al*, 1996) have shown that human lymphocytes express abundant hGH mRNA. These considerations and our present results, suggest that autocrine/paracrine GH and/or PRL in the reactive stroma of ductal carcinoma may be inducers of SOCS gene expression *in vivo*.

In order to establish whether this SOCS overexpression is intrinsic to the transformed breast cancer cell or a result of host–tumour interaction, we also examined 10 breast cancer lines for SOCS gene expression. However, only CIS gene expression was elevated in all of these lines, but not in the two control breast lines. Immunoblots showed a high level of expression of ubiquitinylated CIS or CIS protein in the cancer lines, whereas neither form of CIS was detected in the control line. Level of protein expression of CIS was not affected by serum starvation, suggesting that elevated CIS expression is intrinsic to these breast cancer lines, and does not require serum factors such as hGH, prolactin or colony-stimulating factors. Accordingly, there was no correlation between level of gene expression for either hGH receptor or prolactin receptor and CIS gene expression across these lines.

Given the ubiquitous nature of SOCS gene expression in response to host cytokines, and the above candidate inducers, it is not unexpected that breast tumour tissue shows elevated SOCS expression. However, the elevation of CIS expression is independent of host responses, and appears to be intrinsic to the breast cancer line itself. What might be the significance of this elevation? We suggest that it serves at least a two-fold purpose: (1) to activate the proliferative MAP/ERK kinase pathway and (2) to block STAT 5-mediated responses to trophic hormones, which maintain normal differentiated breast cell phenotype as exemplified by the lack of mammary gland development in CIS transgenic mice (Matsumoto *et al*, 1999). Activation of MAP kinases (ERK, JNK) has been shown to be an important consequence of CIS overexpression in CD4 T cells (Li *et al*, 2000), and is thought to be a result of direct association between CIS and PKC theta, since this PKC is able to activate MAP kinases (Baier-Bitterlich *et al*, 1996). We have confirmed that chronically elevated CIS is able to elevate ERK activity in an ERK responsive promoter assay, and find induction of luciferase reporter activity proportional to the amount of cotransfected CIS expression vector. A coordinated increase in ERK activation and inhibition of STAT5 activation by CIS could allow it to act as a switch, promoting proliferation while blocking differentiated breast cell function. An additional role for the constitutively elevated CIS may be to decrease breast cancer sensitivity to interferons, similar to the decreased sensitivity to IFN*γ* seen in cutaneous T-lymphoma (CTCL) cell lines that constitutively express SOCS-3 (Brender *et al*, 2001).

The alteration responsible for elevated CIS gene and protein expression is unclear, but given that the major factor thought to be responsible for induction of CIS by cytokines is STAT 5 (Verdier *et al*, 1998), the increased CIS expression may be linked to escape from STAT 5-mediated induction of its promoter. This is supported by our preliminary studies that show low or undetectable STAT 5 in the breast cancer lines studied here (Tam, Lau, Waters, unpublished). Other transcription factors have been shown to interact with the CIS promoter such as Ets factors GABP*α*/*β* and NF*κ*B, and deletion of STAT 5 elements in the proximal human CIS promoter still results in substantial levels of basal activity (Verdier *et al*, 1998), so other *cis* acting factors may be important in maintaining CIS expression in these breast cancer cells.

The significance of the strongly elevated level of ubiquitinylated CIS is unclear, but may relate to the ability of CIS to promote destruction of cytokine–cytokine receptor complexes via the ubiquitin-proteasome pathway (Tauchi *et al*, 2001). This has been shown to substantially decrease cell sensitivity to the relevant cytokine (Ram and Waxman, 2000; Tauchi *et al*, 2001).

In conclusion, we report elevation of SOCS-1–3 and CIS in breast cancers *in vivo*. In breast cancer lines, only CIS gene expression was elevated, and this could have important consequences for breast cancer phenotype and proliferative ability. Given this observation, agents blocking the production or action of CIS could be of therapeutic benefit in breast cancer.
